# Synergistic Effects of Essential Oils and Organic Acids against *Aspergillus flavus* Contamination in Poultry Feed

**DOI:** 10.3390/toxins15110635

**Published:** 2023-10-31

**Authors:** Tim Satterlee, Callie Megan McDonough, Scott E. Gold, Chongxiao Chen, Anthony E. Glenn, Anthony Pokoo-Aikins

**Affiliations:** 1 Toxicology & Mycotoxin Research Unit, U.S. National Poultry Research Center, Agricultural Research Service, U.S. Department of Agriculture, 950 College Station Road, Athens, GA 30605, USA; tim.satterlee@usda.gov (T.S.); callie.mcdonough@usda.gov (C.M.M.); scott.gold@usda.gov (S.E.G.);; 2 Department of Poultry Science, University of Georgia, 110 Cedar Street, Athens, GA 30602, USA; sean.chen@uga.edu

**Keywords:** *Aspergillus flavus*, essential oil, organic acid, aflatoxin, postharvest, corn, poultry feed

## Abstract

Organic acids and essential oils are commonly used in the poultry industry as antimicrobials and for their beneficial effects on gut health, growth performance, and meat quality. A common postharvest storage fungal colonist, *Aspergillus flavus*, contaminates corn, the primary component of poultry feed, with the highly detrimental mycotoxin, aflatoxin. Aflatoxin adversely affects poultry feed intake, feed conversion efficiency, weight gain, egg production, fertility, hatchability, and poultry meat yield. Both organic acids and essential oils have been reported to inhibit the growth of *A. flavus.* Thus, we evaluated if the inhibitory synergy between combined essential oils (cinnamon, lemongrass, and oregano) and organic acids (acetic, butyric, and propionic) prevents *A. flavus* growth. The study confirmed that these compounds inhibit the growth of *A. flavus* and that synergistic interactions do occur between some of them. Overall, cinnamon oil was shown to have the highest synergy with all the organic acids tested, requiring 1000 µL/L air of cinnamon oil and 888 mg/kg of butyric acid to fully suppress *A. flavus* growth on corn kernels. With the strong synergism demonstrated, combining certain essential oils and organic acids offers a potentially effective natural method for controlling postharvest aflatoxin contamination in poultry feed.

## 1. Introduction

Aflatoxin, the first compound to cause coinage of the word “mycotoxin”, was identified after a mass turkey die-off in England during the 1960s killing over 100,000 birds [1]. The outbreak was caused by aflatoxin contamination of peanut meal used in the poultry feed formulation. Limited options exist to mitigate the effects of mycotoxins in poultry feed once consumed; therefore, prevention is the best method to avoid aflatoxin contamination and its harmful effects. Prevention of aflatoxin contamination in the feed supply chain is achieved through numerous pre- and post-harvest practices. Among them, the use of Good Agricultural Practices is crucial, such as the proper use of insecticides and antifungals, crop rotation, use of non-aflatoxigenic biocontrol fungal strains, and proper harvest times [2,3]. Because it is impossible to prevent fungal spore contamination from the field, post-harvest practices are necessary to ensure a safe food and feed supply. Currently, there is a demand for the use of natural, safe, and environmentally friendly approaches to reduce fungal and mycotoxin contamination in food and feed.

Organic acids are a class of compounds that naturally occur in living organisms and are used in fundamental metabolic processes [4]. Propionic acid, for example, naturally exists in milk products and is produced by *Propionibacteria* [5]. Organic acids are also commonly used in food processing and preservation [6]. Some organic acids, such as citric and lactic acid, can improve the nutritional properties of feed through the degradation of anti-nutritive substances [6]. Butyric acid (BA), when added to a poultry diet, has shown positive effects on health, performance, and can be an effective alternative to antibiotics against *Salmonella* and *Escherichia coli* infections [7]. Numerous studies have shown the efficacy of various organic acids in inhibiting fungal growth and mycotoxin production. Some organic acids inhibit fungal growth, while others allow growth but inhibit the production of mycotoxins. Hassan et al. [8] examined several organic acids and found that while all were able to inhibit the growth of the *A. flavus* strains tested, only acetic, formic, and lactic acid partially inhibited aflatoxin production in addition to reducing growth. An early study showed that both propionic and butyric acids at sublethal doses were able to reduce aflatoxin production in *A. flavus* [9].

Essential oils consist of the major volatile secondary metabolic products isolated from leaves, bark, flowers, buds, stems, roots, or fruit of aromatic plants [10]. These metabolic products include alcohols, ketones, aldehydes, terpenes, and other characteristic compounds [11]. Essential oils are known for their antioxidant, antimicrobial, analgesic, antipyretic, antiulcer, anticonvulsant, and anticancer properties [12]. Essential oils also improve poultry growth performance and control the growth of intestinal microflora (e.g., reduce pathogenic bacteria), thus improving gut health and meat quality [13,14]. Essential oils achieve this by stimulating digestive enzyme secretion and stabilizing the gut microflora ecosystem [15,16,17,18,19,20]. Overall, with industrial curtailment of antibiotic use, essential oils are an attractive alternative in poultry production due to their natural, less problematic properties [13,21]. In specific combinations, paired essential oils or an essential oil combined with other decontamination treatments can create synergistic effects [22,23]. Xiang et al. [22] found that cinnamon, oregano, and lemongrass essential oils in a 1:5:48 ratio exhibited synergy when compared to the individual essential oils alone and that combination was able to reduce aflatoxin production by one half via transcriptional repression of the aflatoxin biosynthetic genes.

The beneficial synergy between essential oils and organic acids has been studied before, but only regarding the protection of poultry health against pathogenic bacteria [23,24]. At the time of writing this paper, the combined effectiveness of any organic acid and essential oil has not been tested against *A. flavus.* In the current study, we hypothesized that synergy between organic acids and essential oils will effectively combat postharvest *A. flavus* growth and subsequent aflatoxin contamination in corn destined for poultry feed. To test this, we initially exposed *A. flavus* to three organic acids (acetic acid (AA), butyric acid (BA), and propionic (PA)) and three essential oils (cinnamon oil (CO), lemongrass oil (LO), and oregano oil (OO)) to determine the minimal inhibitory concentration (MIC). For this study, we used the definition of synergy as exceeding the sum of the effects of combined inhibitors [25]. To determine the synergy, sub-inhibitory doses of organic acids and/or essential oils were combined to control the growth of *A. flavus.* Initial studies were conducted in media with additional experiments using corn kernels to simulate the relevant contamination conditions more closely. We demonstrated that specific essential oils provide synergistic inhibition of *A. flavus* growth when combined with organic acids at concentrations well below one-half of their respective MICs.

## 2. Results

### 2.1. Effects of Select Organic Acids and Essential Oils against A. flavus Growth on Potato Dextrose Media

**Figure 1:**
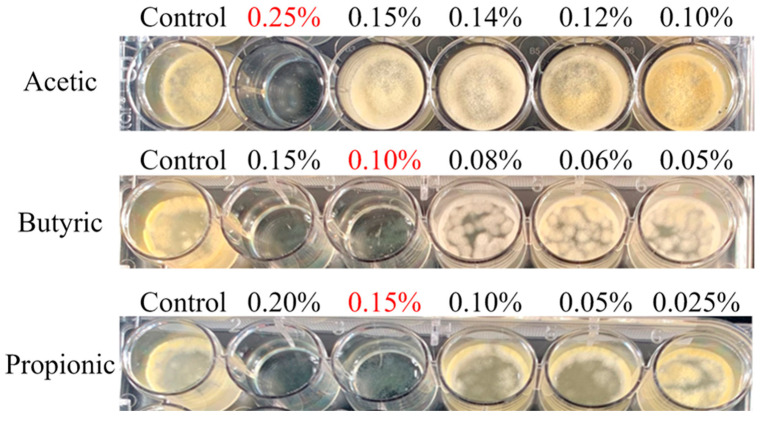
Minimal inhibitory concentrations (MICs) of three organic acids against *Aspergillus flavus*. Cultures were tested in liquid PDB for three days. Each organic acid was added to the indicated final concentration. The red text highlights the concentrations designated as the MIC.

**Figure 2:**
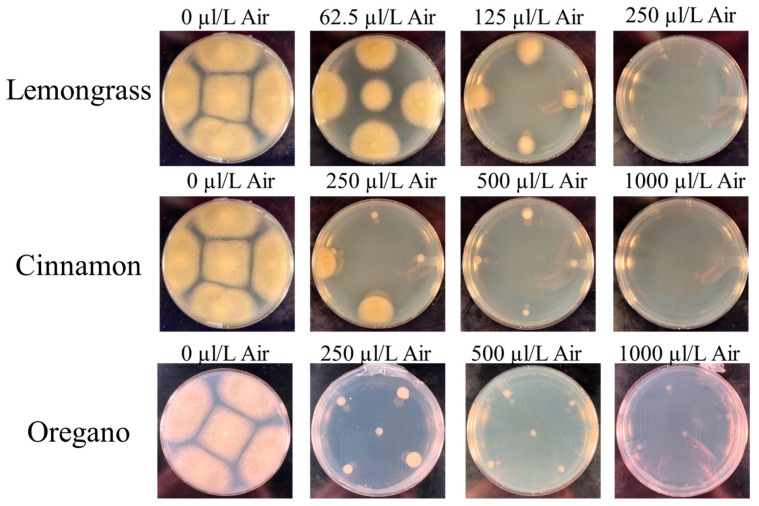
Minimal inhibitory concentration of essential oils against *Aspergillus flavus* on solid PDA. The MIC of LO and CO was determined to be 250 µL/L and 1000 µL/L air, respectively. No MIC for OO was determined because even at 8000 µL/L air, while strongly inhibitory, it did not fully suppress *A. flavus* growth.

Three organic acids (acetic acid (AA), butyric acid (BA), and propionic acid (PA)) were tested in the current study, with BA proving to be the most effective at preventing the growth of *A. flavus* with the lowest MIC at a final concentration of 0.10% (Figure 1). PA was the second most effective at 0.15%, while AA required the highest concentration at 0.25% to fully inhibit *A. flavus* growth (Figure 2). Three essential oils were compared to inhibit *A. flavus* growth (cinnamon oil (CO), lemongrass oil (LO), and oregano oil (OO)). In contrast to the organic acids, a wide range of MICs was observed between the essential oils. LO required 250 µL/L air for its vapors to fully suppress the growth of *A. flavus,* while CO required four times that amount at 1000 µL/L air to achieve a similar result (Figure 2). OO has been previously reported [22] to have antifungal activity against *A. flavus*, which was also observed in this study However, at 32 times (8000 µL/ L air) the MIC of LO, *A. flavus* was still able to grow albeit slowly. Due to this lack of efficacy, OO was eliminated from further synergy testing. To determine if the MIC was fungistatic or fungicidal, cells at the inoculation point were recovered with a sterile cotton swab and streaked on a fresh PDA plate without any essential oils or organic acids added. After three days of incubation, only the LO proved to be fungistatic, producing new growth, while all other tested organic acids and essential oils were fungicidal.

### 2.2. Synergism between Organic Acids and Essential Oils to Inhibit A. flavus Growth on PDA

Based on their individual assessments, we tested the inhibitory synergy against *A. flavus* by combining the most effective organic acid MICs with CO and LO using serial half dilutions (Table 1 and Table 2). We found that all organic acid combinations mixed with 1/2 MIC of either oil caused complete growth inhibition of *A. flavus*. It also appeared that in synergistic interactions, the oil was the major component inhibiting *A. flavus*, as the fungus became more resistant to combinations with lower amounts of oil vs. lower amounts of organic acid (Table 1 and Table 2). This was evident because reducing any organic acid down to 1/16 its MIC, then adding 1/2 the MIC of either CO or LO, demonstrated full inhibition. The inverse combinations reduced *A. flavus* growth, but not fully. On solid PDA plates, PA and LO demonstrated the highest synergy (Table 1). CO showed versatile inhibitory synergy in all organic acid combinations (Table 2).

### 2.3. Effects of Select Organic Acids and Essential Oils agasint A. flavus on Corn Kernels

The MICs for each compound found to be effective against *A. flavus* on PDA were also tested for the inhibitory effects of *A. flauvs* on corn kernels. The results indicated that the MICs observed on agar were not sufficient to inhibit fungal growth on kernels. Based on this result, we repeated our initial experiments to determine the MICs of each compound for corn kernels (Table 3). Compared to our trials on agar, LO performed poorly. Previously, LO outperformed CO at 1/4 of the MIC ratio, but when using kernels, LO was a much weaker inhibitor. Even at 8000 µL/L air, LO was still unable to completely inhibit fungal growth on kernels, whereas CO at that concentration fully inhibited *A. flavus* growth on kernels. In contrast to the essential oils, the three organic acids required relatively similar concentrations (AA = 1500 mg/kg, BA = 1760 mg/kg, and PA = 2222 mg /kg) to fully inhibit *A. flavus* growth.

### 2.4. Synergism between Organic Acids and Essential Oils to Inhibit A. flavus Growth on Corn Kernels

Due to the low efficacy on kernels of OO and LO, we focused further synergy testing on organic acid pairings with CO. In combination with CO, each acid displayed synergy and was able to be used at 1/2 of the MIC and still fully inhibit *A. flavus* growth (Table 4). For example, AA and CO completely suppressed the growth of *A. flavus* at 1/2 MIC (750 mg/kg) of AA and 1/4 MIC (2000 µL/L air) of CO. Further, AA appears to have the weakest synergy with CO since the other tested combinations of CO + AA only partially suppressed, or completely failed to suppress, *A. flavus* growth. BA and PA showed similar synergestic results with CO. Combining 1/2 MIC for either organic acid (BA = 880 mg/kg, PA = 1111 mg/kg) with 1/4 MIC of CO (1000 µL/L air) completely prevented *A. flavus* growth on kernels. When lowering the organic acid concentration below 1/2, the MIC resulted in only partial growth inhibition. All other combinations tested did not fully suppress the growth of *A. flavus* growth on corn kernels.

## 3. Discussion

To prevent *A. flavus* contamination and subsequent aflatoxin contamination, both organic acids and essential oils have been previously studied, albeit separately in poultry feed. As agricultural antimicrobial drug use has become more restricted in developed nations, the industry is pushing for more natural solutions as mycotoxin contamination preventatives. The end goal of this research is to find a cost-effective treatment to prevent aflatoxin contamination in poultry feed. The intial pH values of broiler and layer feeds are between 6.1 and 7.2 [26]. Many previous studies have demonstrated that more acidic condtions (between pH 5.0 and 5.5) are more conducive to *A. flavus* growth and to its ability to produce aflatoxin [27,28,29]. The use of organic acids is not a novel concept for avoiding microbial contamination and it is used to suppress *Salmonella* and *Clostridum* bacteria [30,31]. If used to prevent the spread of *A. flavus,* these acids would likely decrease the pH of the feed. Therefore, not fully eliminating the fungus would potentially create conditions more conducive to the production of aflatoxin. As seen in Figure 1, the lowest concentration of acetic acid facilitated better growth than the control, consistent with the better growth of *A. flavus* at a lower pH. While synergy between different essential oils has been found before [22], this study is the first to examine combining essential oils and organic acids together to combat *A. flavus* growth and thus prevent aflatoxin contamination.

Testing essential oils and organic acids together has revealed synergy in their inhibition of *A. flavus.* The results demonstrated that the levels of synergy varied between chemical combinations, with higher concentrations needed to control growth on kernels than on synthetic media. On PDA, the combination of LO and PA was the most effective pairing requiring the lowest doses to fully inhibit *A. flavus*. CO and the other two organic acids also performed well, but required higher concentrations. In contrast, testing MIC concentrations from PDA on corn kernels revealed large differences in performance with LO particularly underperforming. Synergy on kernels was confirmed between CO and all three organic acids, even though the original MICs determined on agar were unable to inhibit *A. flavus* growth on corn. In *A. flavus* infections, the fungus will penetrate the outer wall of the kernel to reach the endospore of the seed and obtain nutrients [32]. Since LO was only mildly inhibitory on corn kernels, it is likely some fungal spores on the surface were able to germinate and infect the kernel. Within the seed, the fungus would have been able to thrive and potentially contaminate using the kernels with aflatoxin. This highlights the fact that while in vitro experimental results can be used to infer relationships for practical purposes, it is important to test conditions that adequately mimic real-world settings.

Previous literature has demonstrated that specific organic acids and essential oils can reduce the growth of *A. flavus* and aflatoxin production [8,9,22,33,34]. Additional studies also revealed that these compounds supressed the expression of genes in the aflatoxin gene cluster including *aflR* and the global regulatory gene *laeA* [22,35,36]. However, in these studies, while severely reduced upon exposure to these compounds at sublethal doses, the fungus was still able to produce aflatoxin albeit at a reduced capacity. This indicates that if *A. flavus* growth is not fully inhibited, it could eventually produce a dangerous amount of aflatoxin. Additionally, while aflatoxin is the only regulated mycotoxin that *A. flavus* produces, it can produce others, such as aflatrem, a tremorgenic mycotoxin, and cyclopiazonic acid, an indole-tetramic mycotoxin that is an inhibitor of calcium dependent-ATPases [37,38]. Aflatrem has not been studied in poultry but in rats, aflatrem was shown to decrease the capacity of GABA and glutamate uptake systems affecting nuerotransmitters and also potentially causing nerve terminal degradation [39]. In broilers, cyclopiazonic acid was shown to cause ulcerative proventriculitis, gizzard mucosal necrosis, and hepatic and splenic inflammation and necrosis [40]. Since *A. flavus* in most cases was not killed, when these compounds eventually dissipate, the fungus will likely contaminate food and feed with the aforementioned mycotoxins. Research into the duration that these organic acids and essential oils are inhibitory requires further study. To avoid this problem, our current study focused on determining the concentrations that were fungicidal and completely stopped the growth of *A. flavus.*

As stated earlier, this is the first study to combine essential oils and organic acids and test their effectiveness in *A. flavus* growth inhibition, but this is not the first time the synergies between organic acids and essential oils have been evaluated. Yang et al. [24] found that mixtures of essential oils and organic acids improved feed conversion ratios through their antimicrobial activities and by promoting digestive enzyme function. Pham et al. [23] detailed the beneficial effects of using encapsulated essential oils and organic acids in poultry feed, which improved growth performance, modulated cecal microbiota, and had the potential to be an antimicrobial alternative with a focus on mitigating necrotic enteritis gut impairment. Necrotic enteritis is a major economic problem affecting poultry health and productivity [41,42,43]. While not a direct cause, ingested aflatoxin has been shown to predispose birds to necrotic enteritis [44]. This suggests that applying effective combinations of essential oils and organic acids to poultry feed has the potential to both combat mycotoxin contamination and improve the overall health and performance of poultry.

## 4. Conclusions

As industries push toward the use of more natural antimicrobials, essential oils and organic acids offer an alternative to combat *A. flavus* growth and subsequent aflatoxin contamination. The poultry industry has already started using these compounds for their health benefits in birds and their inhibitory effect on pathogenic bacteria. Our study reconfirmed that these compounds could inhibit the growth of *A. flavus*, but we have also demonstrated that combining organic acids and essential oils offers synergistic effects. For example, we found that using 1/4 of the MIC of CO with 1/2 of the MIC of BA fully inhibited *A. flavus,* demonstrating synergy between these compounds. The results presented here demonstrate that combining selected essential oils and organic acids could be an effective alternative in combating *A. flavus* growth in poultry feed.

## 5. Materials and Methods

### 5.1. Strain and Growth Conditions

In all experiments, the *A. flavus* wild-type strain NRRL3357 was used. *A. flavus* was grown in the dark at 28 °C for all experiments. For experiments using media, solid potato dextrose agar (PDA, Acumedia) or liquid potato dextrose broth (PDB, Acumedia) was used. For inoculum, cultures were grown for 3 days on PDA, washed with sterile water to collect conidia, and then filtered through cheesecloth. Conidia were quantified by hemacytometer.

### 5.2. Minimal Inhibitory Concentration Determination of Organic Acids and Essential Oils

To determine the MIC of organic acids to inhibit *A. flavus* growth, 1 mL of PDB was added to each well of a 24-well plate and specified amounts of pure acetic, butyric, or propionic acid (Millipore Sigma: AA—W200611-10KG-K, BA—W222119-5KG-K, PA—W292400-10KG-K) added. For inoculum, 10^4^ conidia of *A. flavus* were added to each well. Cultures were incubated stationary for 3 days as stated above, and growth was assessed.

Concentrations for each volatile essential oil (cinnamon: Millipore Sigma (#=W22902-1KG-K); lemongrass: Fisher Scientific (8007-02-1); and oregano: Dosto) were based on the unit µL/L air, as described in the work by Císarová et al. [45]. For inoculation, *A. flavus* conidia at a concentration of 5 × 10^3^ suspended in 5 µL of water were spotted at the center and four cardinal points of each plate. Due to the volatile producing nature of essential oils, they were applied to Whatman 2 paper filter discs (42.5 mm) instead of incorporation in the PDA. Filter discs were placed on the lids of the Petri plates, wrapped in parafilm, and incubated inverted. After five days, the cultures were assessed for growth. The lowest concentration that completely inhibited all visible growth was the MIC. Each organic acid and essential oil were tested individually.

### 5.3. Synergy Testing on PDA

**Figure 3:**
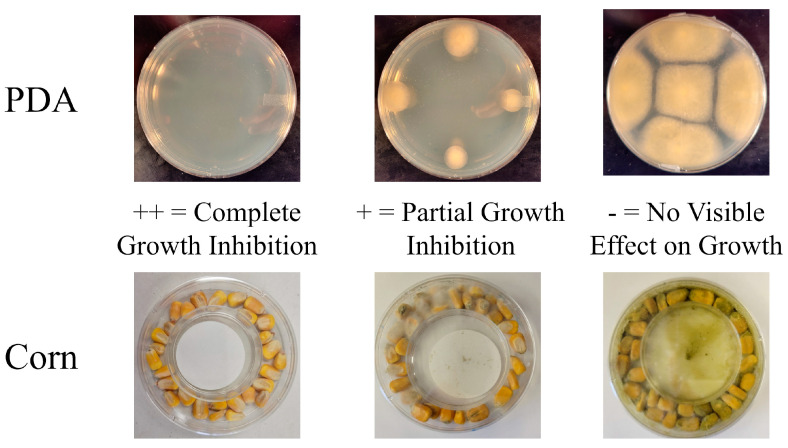
Methodology for essential oil and organic acid scoring for minimal inhibitory concentration and synergy in inhibition of *Aspergillus flavus* growth. Images showing visual cues for scoring full inhibition (++), partial inhibitoni (+), and no inhibition (-) of *A. flavus* in growth experiments.

Synergy tests between essential oils and organic acids combined serial half dilutions (e.g., 1/2, 1/4, 1/8, and 1/16) from each respective MIC. The growth of *A. flavus* was assayed on PDA using these combinations. Appropriate concentrations of each organic acid were added to molten PDA and the volatile essential oils were added on filter paper as described above. Spot inoculation with 5 × 10^3^ conidia of *A. flavus* was performed as described above for testing the essential oil MIC. To identify synergistic combinations, we employed a three-tier (++, +, −) growth scoring system compared to a no-treatment control (Figure 3) with “++” indicating full inhibition of fungal growth, “+” partial inhibition of fungal growth, and “−” for no visible inhibition of fungal growth.

### 5.4. MIC and Synergy Testing on Corn Kernels

Corn kernels were initially screened for damage and only whole, undamaged kernels were used. Kernels underwent a surface and internal disinfestation treatment [46]. Briefly, kernels were washed three times with sterile distilled water, then submerged in a 10% bleach solution for 10 min with moderate agitation followed by another sterile water rinse. This process was repeated with 95% ethanol. To remove internal contaminants, kernels were placed in a 65 °C water bath for 30 min. Kernels were then air dried in a biosafety cabinet and used immediately or maintained in sterile conditions at 4 °C for up to 24 h prior to use.

For individual MIC testing, 10 g (±0.2 g) of sterilized kernels were used for each treatment replicate. Specific amounts of each organic acid were added to 5 mL of sterile water. The dilute organic acid mix was sprayed onto the kernels with an Equate Fingertip Sprayer (59 mL). Kernels were then allowed to air dry in a biosafety hood prior to inoculation. As with agar, the essential oils were placed on a filter disk, which was then placed on the lid of a 60 mm Petri plate. This lid was inverted and placed within a 100 mm standard Petri plate (Figure 3). To simulate infection caused naturally in storage conditions, two *A. flavus* colonized kernels were mixed with the treated seeds. *A. flavus* colonized kernels were produced by placing sterilized kernels in 10 mL of water with conidia (10^6^ conidia/mL) in a petri plate overnight at 28 °C in the dark. Inoculated kernels were transferred to a new plate and incubated for seven days. Treated and infected kernels were then vigorously hand shaken together in a 50 mL conical tube for 30 s and then plated. For all treatment types, kernels were incubated in the dark for five days before assessing fungal growth. As before, a rating scale of “++”, “+”, or “−” was assigned to the treatments as a measure of inhibition. The lowest dose with a “++” rating was determined to be the MIC for that respective compound. Synergy between organic acids and essential oils was tested with a two-fold dilution series (1/2, 1/4, & 1/8) of each compound based on its previously determined MIC on kernels.

## Figures and Tables

**Table 1:** Lemongrass oil synergy with organic acids on solid PDA.

		Lemongrass Oil			
		1/2 MIC	1/4 MIC	1/8 MIC	1/16 MIC
**Acetic Acid**	**1/2 MIC**	++	++	++	+
	**1/4 MIC**	++	+	+	+
	**1/8 MIC**	++	+	−	−
	**1/16 MIC**	++	−	−	−
**Butyric Acid**	**1/2 MIC**	++	++	++	+
	**1/4 MIC**	++	+	+	+
	**1/8 MIC**	++	+	−	−
	**1/16 MIC**	++	+	−	−
**Propionic Acid**	**1/2 MIC**	++	++	++	+
	**1/4 MIC**	++	++	+	+
	**1/8 MIC**	++	++	+	+
	**1/16 MIC**	++	+	+	+

Note: Each combination’s rating was based on its level of inhibition: “++” indicates complete inhibition; “+” indicates partial inhibition; “−” indicates poor or no inhibition. The lowest concentration for a combination that received the “++” rating was designated its combined MIC. Dilutions were based on the full MICs of each compound: lemongrass oil (250 μL/L air), acetic acid (1500 mg/kg), butyric acid (1760 mg/kg), propionic acid (2222 mg/kg).

**Table 2:** Cinnamon oil synergy with organic acids on solid PDA.

		Cinnamon Oil			
		1/2 MIC	1/4 MIC	1/8 MIC	1/16 MIC
**Acetic Acid**	**1/2 MIC**	++	++	+	+
	**1/4 MIC**	++	+	+	+
	**1/8 MIC**	++	+	+	+
	**1/16 MIC**	++	+	+	+
**Butyric Acid**	**1/2 MIC**	++	++	++	+
	**1/4 MIC**	++	+	+	+
	**1/8 MIC**	++	+	+	+
	**1/16 MIC**	++	+	+	+
**Propionic Acid**	**1/2 MIC**	++	++	++	+
	**1/4 MIC**	++	+	+	+
	**1/8 MIC**	++	+	+	+
	**1/16 MIC**	++	+	+	+

Note: Each combination’s rating was based on its level of inhibition: “++” indicates complete inhibition; “+” indicates partial inhibition. The lowest concentration for a combination that received the “++” rating was designated its combined MIC. Dilutions were based on the full MICs of each compound: cinnamon (8000 µL/L air), acetic acid (1500 mg/kg), butyric acid (1760 mg/kg), propionic acid (2222 mg/kg).

**Table 3:** Minimal inhibitory concentration of essential oils and organic acids on corn kernels.

**Cinnamon**	8000 µL/L air	4000 µL/L air	2000 µL/L air	1000 µL/L air
	++	+	+	+
**Lemongrass**	8000 µL/L air	4000 µL/L air	2000 µL/L air	1000 µL/L air
	+	+	+	−
**Acetic Acid**	3000 mg/kg	1500 mg/kg	750 mg/kg	375 mg/kg
	++	++	+	−
**Butyric Acid**	1760 mg/kg	880 mg/kg	440 mg/kg	220 mg/kg
	++	+	+	+
**Propionic Acid**	2222 mg/kg	1111 mg/kg	556 mg/kg	278 mg/kg
	++	+	+	+

Note: A “−” means concentration had no observable effect on the growth of *A. flavus,* a “+” indicates partial growth inhibition, and a “++” rating indicates complete fungal inhibition resulting in no growth. The lowest concentration that received the “++” was designated the MIC.

**Table 4:** Cinnamon oil synergy with organic acids on corn kernels.

		Cinnamon Oil		
		1/2 MIC	1/4 MIC	1/8 MIC
**Acetic Acid**	**1/2 MIC**	++	++	++
	**1/4 MIC**	++	+	+
	**1/8 MIC**	++	+	−
**Butyric Acid**	**1/2 MIC**	++	++	++
	**1/4 MIC**	++	+	+
	**1/8 MIC**	++	+	−
**Propionic Acid**	**1/2 MIC**	++	++	++
	**1/4 MIC**	++	++	+
	**1/8 MIC**	++	+	+

Note: Each combination’s rating was based on its level of inhibition: “++” indicates complete inhibition; “+” indicates partial inhibition; “−” indicates poor or no inhibition. The lowest concentration for a combination that received the “++” rating was designated its combined MIC. Dilutions were based on the full MICs of each compound: cinnamon (8000 µL/L air), acetic acid (1500 mg/kg), butyric acid (1760 mg/kg), propionic acid (2222 mg/kg).

## Data Availability

The data presented in this study are available within this article.
